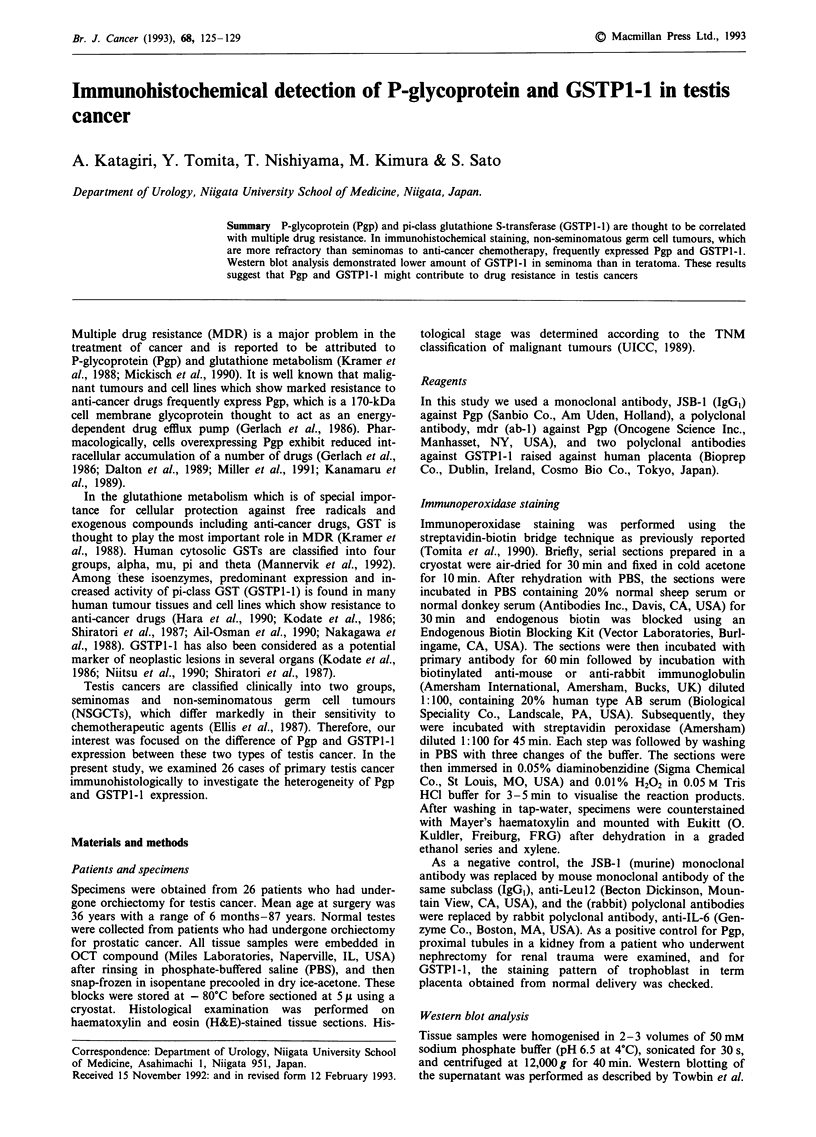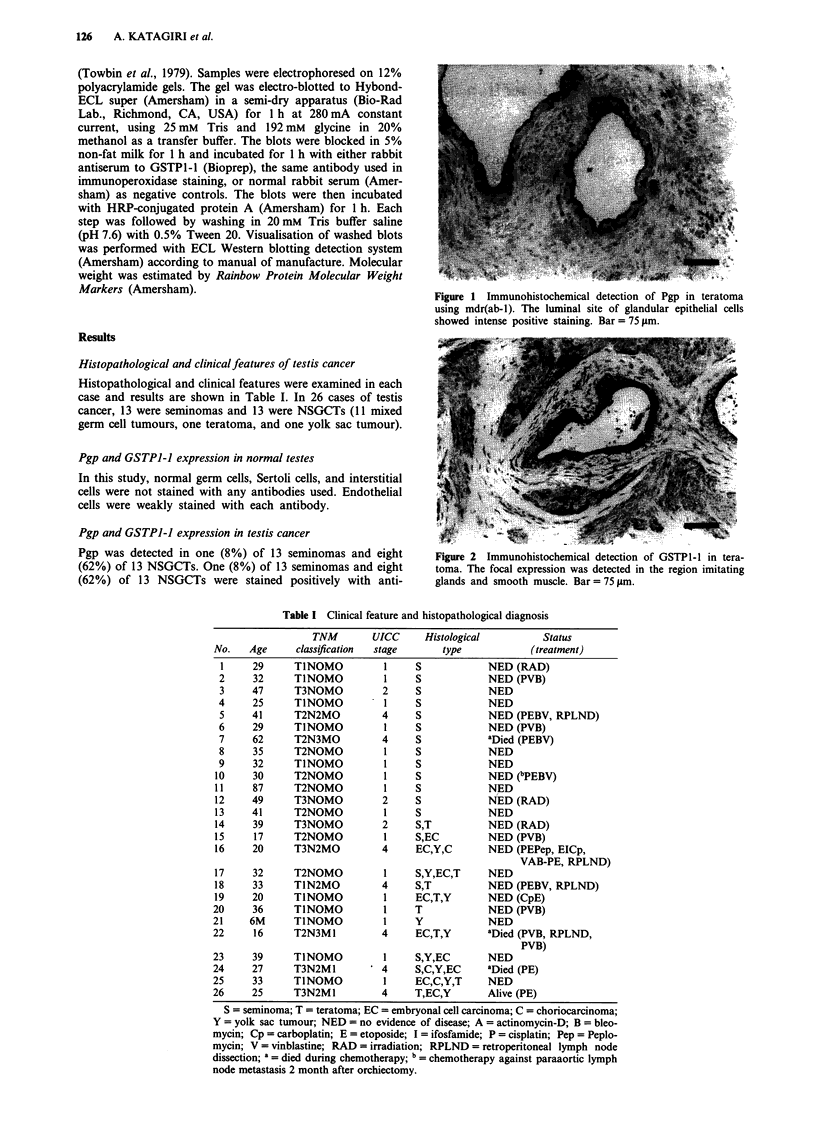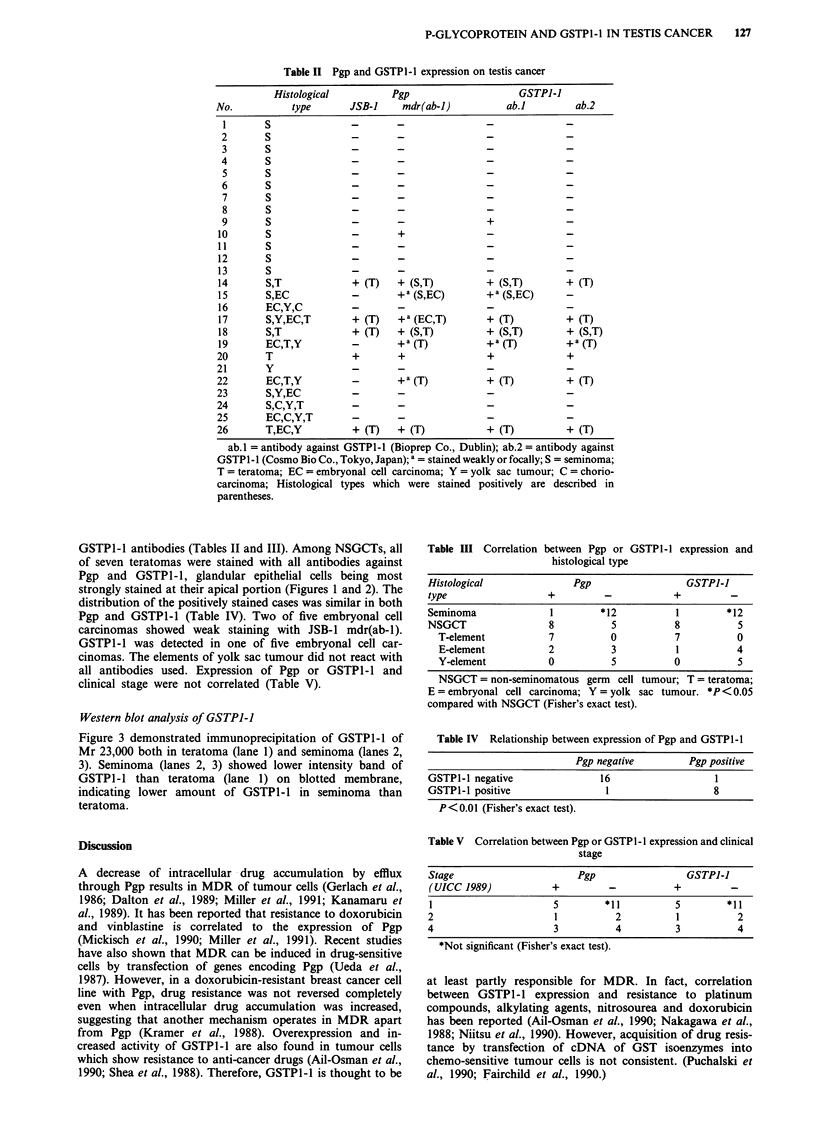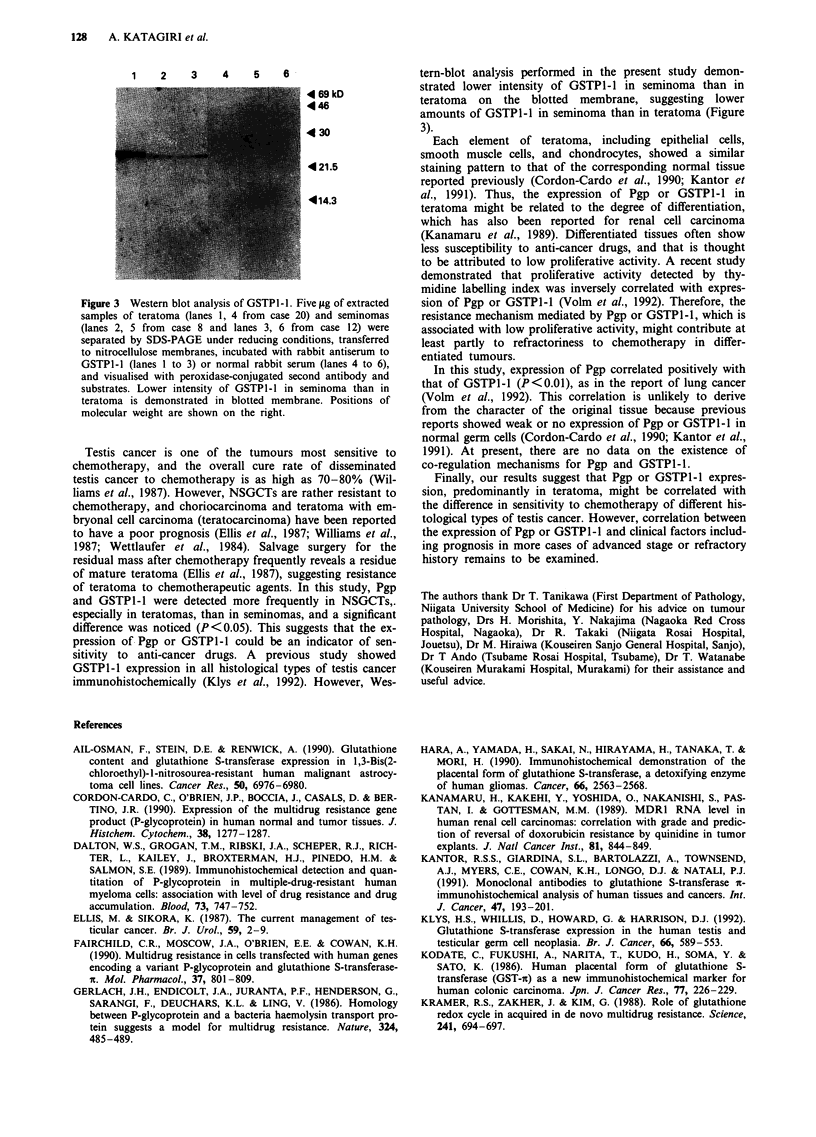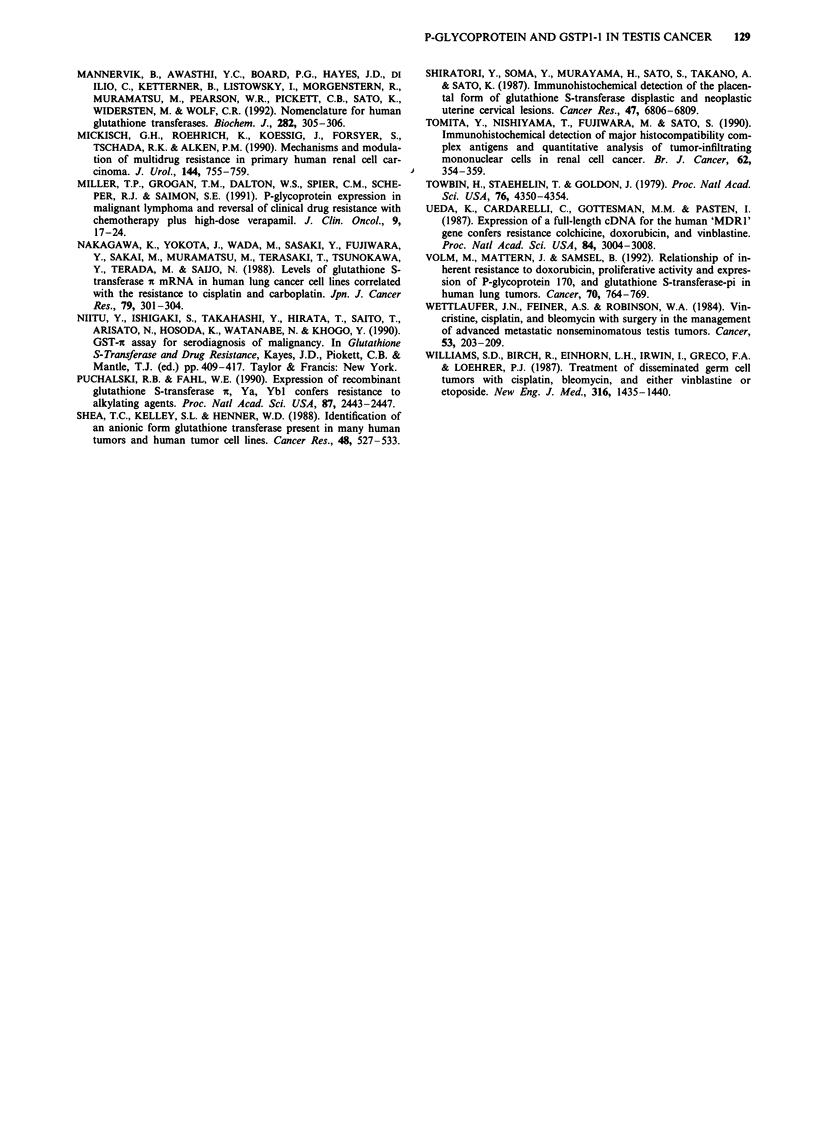# Immunohistochemical detection of P-glycoprotein and GSTP1-1 in testis cancer.

**DOI:** 10.1038/bjc.1993.299

**Published:** 1993-07

**Authors:** A. Katagiri, Y. Tomita, T. Nishiyama, M. Kimura, S. Sato

**Affiliations:** Department of Urology, Niigata University School of Medicine, Japan.

## Abstract

**Images:**


Br. J. Cancer (1993), 68, 125–129 © Macmillan Press Ltd., 1993

### Immunohistochemical detection of P-glycoprotein and GSTP1-1 in testis cancer

A. Katagiri, Y. Tomita, T. Nishiyama, M. Kimura & S. Sato

*Department of Urology, Niigata University School of Medicine, Niigata, Japan.*

**Summary** P-glycoprotein (Pgp) and pi-class glutathione S-transferase (GSTP1-1) are thought to be correlated with multiple drug resistance. In immunohistochemical staining, non-seminomatous germ cell tumours, which are more refractory than seminomas to anti-cancer chemotherapy, frequently expressed Pgp and GSTP1-1. Western blot analysis demonstrated lower amount of GSTP1-1 in seminoma than in teratoma. These results suggest that Pgp and GSTP1-1 might contribute to drug resistance in testis cancers

Multiple drug resistance (MDR) is a major problem in the treatment of cancer and is reported to be attributed to P-glycoprotein (Pgp) and glutathione metabolism (Kramer *et al.*, 1988; Mickisch *et al.*, 1990). It is well known that malignant tumours and cell lines which show marked resistance to anti-cancer drugs frequently express Pgp, which is a 170-kDa cell membrane glycoprotein thought to act as an energy-dependent drug efflux pump (Gerlach *et al.*, 1986). Pharmacologically, cells overexpressing Pgp exhibit reduced intracellular accumulation of a number of drugs (Gerlach *et al.*, 1986; Dalton *et al.*, 1989; Miller *et al.*, 1991; Kanamaru *et al.*, 1989).

In the glutathione metabolism which is of special importance for cellular protection against free radicals and exogenous compounds including anti-cancer drugs, GST is thought to play the most important role in MDR (Kramer *et al.*, 1988). Human cytosolic GSTs are classified into four groups, alpha, mu, pi and theta (Mannervik *et al.*, 1992). Among these isoenzymes, predominant expression and increased activity of pi-class GST (GSTP1-1) is found in many human tumour tissues and cell lines which show resistance to anti-cancer drugs (Hara *et al.*, 1990; Kodate *et al.*, 1986; Shiratori *et al.*, 1987; Ail-Osman *et al.*, 1990; Nakagawa *et al.*, 1988). GSTP1-1 has also been considered as a potential marker of neoplastic lesions in several organs (Kodate *et al.*, 1986; Niitsu *et al.*, 1990; Shiratori *et al.*, 1987).

Testis cancers are classified clinically into two groups, seminomas and non-seminomatous germ cell tumours (NSGCTs), which differ markedly in their sensitivity to chemotherapeutic agents (Ellis *et al.*, 1987). Therefore, our interest was focused on the difference of Pgp and GSTP1-1 expression between these two types of testis cancer. In the present study, we examined 26 cases of primary testis cancer immunohistologically to investigate the heterogeneity of Pgp and GSTP1-1 expression.

### Materials and methods

### *Patients and specimens*

Specimens were obtained from 26 patients who had undergone orchiectomy for testis cancer. Mean age at surgery was 36 years with a range of 6 months–87 years. Normal testes were collected from patients who had undergone orchiectomy for prostatic cancer. All tissue samples were embedded in OCT compound (Miles Laboratories, Naperville, IL, USA) after rinsing in phosphate-buffered saline (PBS), and then snap-frozen in isopentane precooled in dry ice-acetone. These blocks were stored at $-80°C$ before sectioned at $5\,\mu$ using a cryostat. Histological examination was performed on haematoxylin and eosin (H&E)-stained tissue sections. Histological stage was determined according to the TNM classification of malignant tumours (UICC, 1989).

Correspondence: Department of Urology, Niigata University School of Medicine, Asahimachi 1, Niigata 951, Japan.
Received 15 November 1992: and in revised form 12 February 1993.

### *Reagents*

In this study we used a monoclonal antibody, JSB-1 ($IgG_1$) against Pgp (Sanbio Co., Am Uden, Holland), a polyclonal antibody, mdr (ab-1) against Pgp (Oncogene Science Inc., Manhasset, NY, USA), and two polyclonal antibodies against GSTP1-1 raised against human placenta (Bioprep Co., Dublin, Ireland, Cosmo Bio Co., Tokyo, Japan).

### *Immunoperoxidase staining*

Immunoperoxidase staining was performed using the streptavidin-biotin bridge technique as previously reported (Tomita *et al.*, 1990). Briefly, serial sections prepared in a cryostat were air-dried for 30 min and fixed in cold acetone for 10 min. After rehydration with PBS, the sections were incubated in PBS containing 20% normal sheep serum or normal donkey serum (Antibodies Inc., Davis, CA, USA) for 30 min and endogenous biotin was blocked using an Endogenous Biotin Blocking Kit (Vector Laboratories, Burlingame, CA, USA). The sections were then incubated with primary antibody for 60 min followed by incubation with biotinylated anti-mouse or anti-rabbit immunoglobulin (Amersham International, Amersham, Bucks, UK) diluted 1:100, containing 20% human type AB serum (Biological Speciality Co., Landscale, PA, USA). Subsequently, they were incubated with streptavidin peroxidase (Amersham) diluted 1:100 for 45 min. Each step was followed by washing in PBS with three changes of the buffer. The sections were then immersed in 0.05% diaminobenzidine (Sigma Chemical Co., St Louis, MO, USA) and 0.01% $H_2O_2$ in 0.05 M Tris HCl buffer for 3–5 min to visualise the reaction products. After washing in tap-water, specimens were counterstained with Mayer's haematoxylin and mounted with Eukitt (O. Kuldler, Freiburg, FRG) after dehydration in a graded ethanol series and xylene.

As a negative control, the JSB-1 (murine) monoclonal antibody was replaced by mouse monoclonal antibody of the same subclass ($IgG_1$), anti-Leu12 (Becton Dickinson, Mountain View, CA, USA), and the (rabbit) polyclonal antibodies were replaced by rabbit polyclonal antibody, anti-IL-6 (Genzyme Co., Boston, MA, USA). As a positive control for Pgp, proximal tubules in a kidney from a patient who underwent nephrectomy for renal trauma were examined, and for GSTP1-1, the staining pattern of trophoblast in term placenta obtained from normal delivery was checked.

### *Western blot analysis*

Tissue samples were homogenised in 2–3 volumes of 50 mM sodium phosphate buffer (pH 6.5 at 4°C), sonicated for 30 s, and centrifuged at 12,000 *g* for 40 min. Western blotting of the supernatant was performed as described by Towbin *et al.*

(Towbin et al., 1979). Samples were electrophoresed on 12% polyacrylamide gels. The gel was electro-blotted to Hybond-ECL super (Amersham) in a semi-dry apparatus (Bio-Rad Lab., Richmond, CA, USA) for 1 h at 280 mA constant current, using 25 mM Tris and 192 mM glycine in 20% methanol as a transfer buffer. The blots were blocked in 5% non-fat milk for 1 h and incubated for 1 h with either rabbit antiserum to GSTP1-1 (Bioprep), the same antibody used in immunoperoxidase staining, or normal rabbit serum (Amersham) as negative controls. The blots were then incubated with HRP-conjugated protein A (Amersham) for 1 h. Each step was followed by washing in 20 mM Tris buffer saline (pH 7.6) with 0.5% Tween 20. Visualisation of washed blots was performed with ECL Western blotting detection system (Amersham) according to manual of manufacture. Molecular weight was estimated by *Rainbow Protein Molecular Weight Markers* (Amersham).

### Results

### *Histopathological and clinical features of testis cancer*

Histopathological and clinical features were examined in each case and results are shown in Table I. In 26 cases of testis cancer, 13 were seminomas and 13 were NSGCTs (11 mixed germ cell tumours, one teratoma, and one yolk sac tumour).

### *Pgp and GSTP1-1 expression in normal testes*

In this study, normal germ cells, Sertoli cells, and interstitial cells were not stained with any antibodies used. Endothelial cells were weakly stained with each antibody.

### *Pgp and GSTP1-1 expression in testis cancer*

Pgp was detected in one (8%) of 13 seminomas and eight (62%) of 13 NSGCTs. One (8%) of 13 seminomas and eight (62%) of 13 NSGCTs were stained positively with anti-

**Figure 1** Immunohistochemical detection of Pgp in teratoma using mdr(ab-1). The luminal site of glandular epithelial cells showed intense positive staining. Bar = 75 μm.

**Figure 2** Immunohistochemical detection of GSTP1-1 in teratoma. The focal expression was detected in the region imitating glands and smooth muscle. Bar = 75 μm.

**Table I** Clinical feature and histopathological diagnosis

| *No.* | *Age* | *TNM classification* | *UICC stage* | *Histological type* | *Status (treatment)* |
|---|---|---|---|---|---|
| 1 | 29 | T1N0M0 | 1 | S | NED (RAD) |
| 2 | 32 | T1N0M0 | 1 | S | NED (PVB) |
| 3 | 47 | T3N0M0 | 2 | S | NED |
| 4 | 25 | T1N0M0 | 1 | S | NED |
| 5 | 41 | T2N2M0 | 4 | S | NED (PEBV, RPLND) |
| 6 | 29 | T1N0M0 | 1 | S | NED (PVB) |
| 7 | 62 | T2N3M0 | 4 | S | [a]Died (PEBV) |
| 8 | 35 | T2N0M0 | 1 | S | NED |
| 9 | 32 | T1N0M0 | 1 | S | NED |
| 10 | 30 | T2N0M0 | 1 | S | NED ([b]PEBV) |
| 11 | 87 | T2N0M0 | 1 | S | NED |
| 12 | 49 | T3N0M0 | 2 | S | NED (RAD) |
| 13 | 41 | T2N0M0 | 1 | S | NED |
| 14 | 39 | T3N0M0 | 2 | S,T | NED (RAD) |
| 15 | 17 | T2N0M0 | 1 | S,EC | NED (PVB) |
| 16 | 20 | T3N2M0 | 4 | EC,Y,C | NED (PEPep, EICp, VAB-PE, RPLND) |
| 17 | 32 | T2N0M0 | 1 | S,Y,EC,T | NED |
| 18 | 33 | T1N2M0 | 4 | S,T | NED (PEBV, RPLND) |
| 19 | 20 | T1N0M0 | 1 | EC,T,Y | NED (CpE) |
| 20 | 36 | T1N0M0 | 1 | T | NED (PVB) |
| 21 | 6M | T1N0M0 | 1 | Y | NED |
| 22 | 16 | T2N3M1 | 4 | EC,T,Y | [a]Died (PVB, RPLND, PVB) |
| 23 | 39 | T1N0M0 | 1 | S,Y,EC | NED |
| 24 | 27 | T3N2M1 | 4 | S,C,Y,EC | [a]Died (PE) |
| 25 | 33 | T1N0M0 | 1 | EC,C,Y,T | NED |
| 26 | 25 | T3N2M1 | 4 | T,EC,Y | Alive (PE) |

S = seminoma; T = teratoma; EC = embryonal cell carcinoma; C = choriocarcinoma; Y = yolk sac tumour; NED = no evidence of disease; A = actinomycin-D; B = bleomycin; Cp = carboplatin; E = etoposide; I = ifosfamide; P = cisplatin; Pep = Peplomycin; V = vinblastine; RAD = irradiation; RPLND = retroperitoneal lymph node dissection; [a] = died during chemotherapy; [b] = chemotherapy against paraaortic lymph node metastasis 2 month after orchiectomy.

**Table II** Pgp and GSTP1-1 expression on testis cancer

| No. | *Histological type* | *Pgp JSB-1* | *Pgp mdr(ab-1)* | *GSTP1-1 ab.1* | *GSTP1-1 ab.2* |
|---|---|---|---|---|---|
| 1 | S | – | – | – | – |
| 2 | S | – | – | – | – |
| 3 | S | – | – | – | – |
| 4 | S | – | – | – | – |
| 5 | S | – | – | – | – |
| 6 | S | – | – | – | – |
| 7 | S | – | – | – | – |
| 8 | S | – | – | – | – |
| 9 | S | – | – | + | – |
| 10 | S | – | + | – | – |
| 11 | S | – | – | – | – |
| 12 | S | – | – | – | – |
| 13 | S | – | – | – | – |
| 14 | S,T | + (T) | + (S,T) | + (S,T) | + (T) |
| 15 | S,EC | – | +[a] (S,EC) | +[a] (S,EC) | – |
| 16 | EC,Y,C | – | – | – | – |
| 17 | S,Y,EC,T | + (T) | +[a] (EC,T) | + (T) | + (T) |
| 18 | S,T | + (T) | + (S,T) | + (S,T) | + (S,T) |
| 19 | EC,T,Y | – | +[a] (T) | +[a] (T) | +[a] (T) |
| 20 | T | + | + | + | + |
| 21 | Y | – | – | – | – |
| 22 | EC,T,Y | – | +[a] (T) | + (T) | + (T) |
| 23 | S,Y,EC | – | – | – | – |
| 24 | S,C,Y,T | – | – | – | – |
| 25 | EC,C,Y,T | – | – | – | – |
| 26 | T,EC,Y | + (T) | + (T) | + (T) | + (T) |

ab.1 = antibody against GSTP1-1 (Bioprep Co., Dublin); ab.2 = antibody against GSTP1-1 (Cosmo Bio Co., Tokyo, Japan); [a] = stained weakly or focally; S = seminoma; T = teratoma; EC = embryonal cell carcinoma; Y = yolk sac tumour; C = choriocarcinoma; Histological types which were stained positively are described in parentheses.

GSTP1-1 antibodies (Tables II and III). Among NSGCTs, all of seven teratomas were stained with all antibodies against Pgp and GSTP1-1, glandular epithelial cells being most strongly stained at their apical portion (Figures 1 and 2). The distribution of the positively stained cases was similar in both Pgp and GSTP1-1 (Table IV). Two of five embryonal cell carcinomas showed weak staining with JSB-1 mdr(ab-1). GSTP1-1 was detected in one of five embryonal cell carcinomas. The elements of yolk sac tumour did not react with all antibodies used. Expression of Pgp or GSTP1-1 and clinical stage were not correlated (Table V).

### *Western blot analysis of GSTP1-1*

Figure 3 demonstrated immunoprecipitation of GSTP1-1 of Mr 23,000 both in teratoma (lane 1) and seminoma (lanes 2, 3). Seminoma (lanes 2, 3) showed lower intensity band of GSTP1-1 than teratoma (lane 1) on blotted membrane, indicating lower amount of GSTP1-1 in seminoma than teratoma.

**Table III** Correlation between Pgp or GSTP1-1 expression and histological type

| *Histological type* | *Pgp* + | *Pgp* – | *GSTP1-1* + | *GSTP1-1* – |
|---|---|---|---|---|
| Seminoma | 1 | *12 | 1 | *12 |
| NSGCT | 8 | 5 | 8 | 5 |
| T-element | 7 | 0 | 7 | 0 |
| E-element | 2 | 3 | 1 | 4 |
| Y-element | 0 | 5 | 0 | 5 |

NSGCT = non-seminomatous germ cell tumour; T = teratoma; E = embryonal cell carcinoma; Y = yolk sac tumour. *$P<0.05$ compared with NSGCT (Fisher's exact test).

**Table IV** Relationship between expression of Pgp and GSTP1-1

| | *Pgp negative* | *Pgp positive* |
|---|---|---|
| GSTP1-1 negative | 16 | 1 |
| GSTP1-1 positive | 1 | 8 |

$P<0.01$ (Fisher's exact test).

**Table V** Correlation between Pgp or GSTP1-1 expression and clinical stage

| *Stage (UICC 1989)* | *Pgp* + | *Pgp* – | *GSTP1-1* + | *GSTP1-1* – |
|---|---|---|---|---|
| 1 | 5 | *11 | 5 | *11 |
| 2 | 1 | 2 | 1 | 2 |
| 4 | 3 | 4 | 3 | 4 |

*Not significant (Fisher's exact test).

### Discussion

A decrease of intracellular drug accumulation by efflux through Pgp results in MDR of tumour cells (Gerlach *et al.*, 1986; Dalton *et al.*, 1989; Miller *et al.*, 1991; Kanamaru *et al.*, 1989). It has been reported that resistance to doxorubicin and vinblastine is correlated to the expression of Pgp (Mickisch *et al.*, 1990; Miller *et al.*, 1991). Recent studies have also shown that MDR can be induced in drug-sensitive cells by transfection of genes encoding Pgp (Ueda *et al.*, 1987). However, in a doxorubicin-resistant breast cancer cell line with Pgp, drug resistance was not reversed completely even when intracellular drug accumulation was increased, suggesting that another mechanism operates in MDR apart from Pgp (Kramer *et al.*, 1988). Overexpression and increased activity of GSTP1-1 are also found in tumour cells which show resistance to anti-cancer drugs (Ail-Osman *et al.*, 1990; Shea *et al.*, 1988). Therefore, GSTP1-1 is thought to be at least partly responsible for MDR. In fact, correlation between GSTP1-1 expression and resistance to platinum compounds, alkylating agents, nitrosourea and doxorubicin has been reported (Ail-Osman *et al.*, 1990; Nakagawa *et al.*, 1988; Niitsu *et al.*, 1990). However, acquisition of drug resistance by transfection of cDNA of GST isoenzymes into chemo-sensitive tumour cells is not consistent. (Puchalski *et al.*, 1990; Fairchild *et al.*, 1990.)

**Figure 3** Western blot analysis of GSTP1-1. Five μg of extracted samples of teratoma (lanes 1, 4 from case 20) and seminomas (lanes 2, 5 from case 8 and lanes 3, 6 from case 12) were separated by SDS-PAGE under reducing conditions, transferred to nitrocellulose membranes, incubated with rabbit antiserum to GSTP1-1 (lanes 1 to 3) or normal rabbit serum (lanes 4 to 6), and visualised with peroxidase-conjugated second antibody and substrates. Lower intensity of GSTP1-1 in seminoma than in teratoma is demonstrated in blotted membrane. Positions of molecular weight are shown on the right.

Testis cancer is one of the tumours most sensitive to chemotherapy, and the overall cure rate of disseminated testis cancer to chemotherapy is as high as 70–80% (Williams *et al.*, 1987). However, NSGCTs are rather resistant to chemotherapy, and choriocarcinoma and teratoma with embryonal cell carcinoma (teratocarcinoma) have been reported to have a poor prognosis (Ellis *et al.*, 1987; Williams *et al.*, 1987; Wettlaufer *et al.*, 1984). Salvage surgery for the residual mass after chemotherapy frequently reveals a residue of mature teratoma (Ellis *et al.*, 1987), suggesting resistance of teratoma to chemotherapeutic agents. In this study, Pgp and GSTP1-1 were detected more frequently in NSGCTs,. especially in teratomas, than in seminomas, and a significant difference was noticed ($P < 0.05$). This suggests that the expression of Pgp or GSTP1-1 could be an indicator of sensitivity to anti-cancer drugs. A previous study showed GSTP1-1 expression in all histological types of testis cancer immunohistochemically (Klys *et al.*, 1992). However, Western-blot analysis performed in the present study demonstrated lower intensity of GSTP1-1 in seminoma than in teratoma on the blotted membrane, suggesting lower amounts of GSTP1-1 in seminoma than in teratoma (Figure 3).

Each element of teratoma, including epithelial cells, smooth muscle cells, and chondrocytes, showed a similar staining pattern to that of the corresponding normal tissue reported previously (Cordon-Cardo *et al.*, 1990; Kantor *et al.*, 1991). Thus, the expression of Pgp or GSTP1-1 in teratoma might be related to the degree of differentiation, which has also been reported for renal cell carcinoma (Kanamaru *et al.*, 1989). Differentiated tissues often show less susceptibility to anti-cancer drugs, and that is thought to be attributed to low proliferative activity. A recent study demonstrated that proliferative activity detected by thymidine labelling index was inversely correlated with expression of Pgp or GSTP1-1 (Volm *et al.*, 1992). Therefore, the resistance mechanism mediated by Pgp or GSTP1-1, which is associated with low proliferative activity, might contribute at least partly to refractoriness to chemotherapy in differentiated tumours.

In this study, expression of Pgp correlated positively with that of GSTP1-1 ($P < 0.01$), as in the report of lung cancer (Volm *et al.*, 1992). This correlation is unlikely to derive from the character of the original tissue because previous reports showed weak or no expression of Pgp or GSTP1-1 in normal germ cells (Cordon-Cardo *et al.*, 1990; Kantor *et al.*, 1991). At present, there are no data on the existence of co-regulation mechanisms for Pgp and GSTP1-1.

Finally, our results suggest that Pgp or GSTP1-1 expression, predominantly in teratoma, might be correlated with the difference in sensitivity to chemotherapy of different histological types of testis cancer. However, correlation between the expression of Pgp or GSTP1-1 and clinical factors including prognosis in more cases of advanced stage or refractory history remains to be examined.

The authors thank Dr T. Tanikawa (First Department of Pathology, Niigata University School of Medicine) for his advice on tumour pathology, Drs H. Morishita, Y. Nakajima (Nagaoka Red Cross Hospital, Nagaoka), Dr R. Takaki (Niigata Rosai Hospital, Jouetsu), Dr M. Hiraiwa (Kouseiren Sanjo General Hospital, Sanjo), Dr T Ando (Tsubame Rosai Hospital, Tsubame), Dr T. Watanabe (Kouseiren Murakami Hospital, Murakami) for their assistance and useful advice.

### References

AIL-OSMAN, F., STEIN, D.E. & RENWICK, A. (1990). Glutathione content and glutathione S-transferase expression in 1,3-Bis(2-chloroethyl)-1-nitrosourea-resistant human malignant astrocytoma cell lines. *Cancer Res.*, **50**, 6976–6980.

CORDON-CARDO, C., O'BRIEN, J.P., BOCCIA, J., CASALS, D. & BERTINO, J.R. (1990). Expression of the multidrug resistance gene product (P-glycoprotein) in human normal and tumor tissues. *J. Histchem. Cytochem.*, **38**, 1277–1287.

DALTON, W.S., GROGAN, T.M., RIBSKI, J.A., SCHEPER, R.J., RICHTER, L., KAILEY, J., BROXTERMAN, H.J., PINEDO, H.M. & SALMON, S.E. (1989). Immunohistochemical detection and quantitation of P-glycoprotein in multiple-drug-resistant human myeloma cells: association with level of drug resistance and drug accumulation. *Blood*, **73**, 747–752.

ELLIS, M. & SIKORA, K. (1987). The current management of testicular cancer. *Br. J. Urol.*, **59**, 2–9.

FAIRCHILD, C.R., MOSCOW, J.A., O'BRIEN, E.E. & COWAN, K.H. (1990). Multidrug resistance in cells transfected with human genes encoding a variant P-glycoprotein and glutathione S-transferase-π. *Mol. Pharmacol.*, **37**, 801–809.

GERLACH, J.H., ENDICOLT, J.A., JURANTA, P.F., HENDERSON, G., SARANGI, F., DEUCHARS, K.L. & LING, V. (1986). Homology between P-glycoprotein and a bacteria haemolysin transport protein suggests a model for multidrug resistance. *Nature*, **324**, 485–489.

HARA, A., YAMADA, H., SAKAI, N., HIRAYAMA, H., TANAKA, T. & MORI, H. (1990). Immunohistochemical demonstration of the placental form of glutathione S-transferase, a detoxifying enzyme of human gliomas. *Cancer*, **66**, 2563–2568.

KANAMARU, H., KAKEHI, Y., YOSHIDA, O., NAKANISHI, S., PASTAN, I. & GOTTESMAN, M.M. (1989). MDR1 RNA level in human renal cell carcinomas: correlation with grade and prediction of reversal of doxorubicin resistance by quinidine in tumor explants. *J. Natl Cancer Inst.*, **81**, 844–849.

KANTOR, R.S.S., GIARDINA, S.L., BARTOLAZZI, A., TOWNSEND, A.J., MYERS, C.E., COWAN, K.H., LONGO, D.J. & NATALI, P.J. (1991). Monoclonal antibodies to glutathione S-transferase π-immunohistochemical analysis of human tissues and cancers. *Int. J. Cancer*, **47**, 193–201.

KLYS, H.S., WHILLIS, D., HOWARD, G. & HARRISON, D.J. (1992). Glutathione S-transferase expression in the human testis and testicular germ cell neoplasia. *Br. J. Cancer*, **66**, 589–553.

KODATE, C., FUKUSHI, A., NARITA, T., KUDO, H., SOMA, Y. & SATO, K. (1986). Human placental form of glutathione S-transferase (GST-π) as a new immunohistochemical marker for human colonic carcinoma. *Jpn. J. Cancer Res.*, **77**, 226–229.

KRAMER, R.S., ZAKHER, J. & KIM, G. (1988). Role of glutathione redox cycle in acquired in de novo multidrug resistance. *Science*, **241**, 694–697.

MANNERVIK, B., AWASTHI, Y.C., BOARD, P.G., HAYES, J.D., DI ILIO, C., KETTERNER, B., LISTOWSKY, I., MORGENSTERN, R., MURAMATSU, M., PEARSON, W.R., PICKETT, C.B., SATO, K., WIDERSTEN, M. & WOLF, C.R. (1992). Nomenclature for human glutathione transferases. *Biochem. J.*, **282**, 305–306.

MICKISCH, G.H., ROEHRICH, K., KOESSIG, J., FORSYER, S., TSCHADA, R.K. & ALKEN, P.M. (1990). Mechanisms and modulation of multidrug resistance in primary human renal cell carcinoma. *J. Urol.*, **144**, 755–759.

MILLER, T.P., GROGAN, T.M., DALTON, W.S., SPIER, C.M., SCHEPER, R.J. & SAIMON, S.E. (1991). P-glycoprotein expression in malignant lymphoma and reversal of clinical drug resistance with chemotherapy plus high-dose verapamil. *J. Clin. Oncol.*, **9**, 17–24.

NAKAGAWA, K., YOKOTA, J., WADA, M., SASAKI, Y., FUJIWARA, Y., SAKAI, M., MURAMATSU, M., TERASAKI, T., TSUNOKAWA, Y., TERADA, M. & SAIJO, N. (1988). Levels of glutathione S-transferase π mRNA in human lung cancer cell lines correlated with the resistance to cisplatin and carboplatin. *Jpn. J. Cancer Res.*, **79**, 301–304.

NIITU, Y., ISHIGAKI, S., TAKAHASHI, Y., HIRATA, T., SAITO, T., ARISATO, N., HOSODA, K., WATANABE, N. & KHOGO, Y. (1990). GST-π assay for serodiagnosis of malignancy. In *Glutathione S-Transferase and Drug Resistance*, Kayes, J.D., Piokett, C.B. & Mantle, T.J. (ed.) pp. 409–417. Taylor & Francis: New York.

PUCHALSKI, R.B. & FAHL, W.E. (1990). Expression of recombinant glutathione S-transferase π, Ya, Yb1 confers resistance to alkylating agents. *Proc. Natl Acad. Sci. USA*, **87**, 2443–2447.

SHEA, T.C., KELLEY, S.L. & HENNER, W.D. (1988). Identification of an anionic form glutathione transferase present in many human tumors and human tumor cell lines. *Cancer Res.*, **48**, 527–533.

SHIRATORI, Y., SOMA, Y., MURAYAMA, H., SATO, S., TAKANO, A. & SATO, K. (1987). Immunohistochemical detection of the placental form of glutathione S-transferase displastic and neoplastic uterine cervical lesions. *Cancer Res.*, **47**, 6806–6809.

TOMITA, Y., NISHIYAMA, T., FUJIWARA, M. & SATO, S. (1990). Immunohistochemical detection of major histocompatibility complex antigens and quantitative analysis of tumor-infiltrating mononuclear cells in renal cell cancer. *Br. J. Cancer*, **62**, 354–359.

TOWBIN, H., STAEHELIN, T. & GOLDON, J. (1979). *Proc. Natl Acad. Sci. USA*, **76**, 4350–4354.

UEDA, K., CARDARELLI, C., GOTTESMAN, M.M. & PASTEN, I. (1987). Expression of a full-length cDNA for the human 'MDR1' gene confers resistance colchicine, doxorubicin, and vinblastine. *Proc. Natl Acad. Sci. USA*, **84**, 3004–3008.

VOLM, M., MATTERN, J. & SAMSEL, B. (1992). Relationship of inherent resistance to doxorubicin, proliferative activity and expression of P-glycoprotein 170, and glutathione S-transferase-pi in human lung tumors. *Cancer*, **70**, 764–769.

WETTLAUFER, J.N., FEINER, A.S. & ROBINSON, W.A. (1984). Vincristine, cisplatin, and bleomycin with surgery in the management of advanced metastatic nonseminomatous testis tumors. *Cancer*, **53**, 203–209.

WILLIAMS, S.D., BIRCH, R., EINHORN, L.H., IRWIN, I., GRECO, F.A. & LOEHRER, P.J. (1987). Treatment of disseminated germ cell tumors with cisplatin, bleomycin, and either vinblastine or etoposide. *New Eng. J. Med.*, **316**, 1435–1440.